# Tanshinol Rescues the Impaired Bone Formation Elicited by Glucocorticoid Involved in KLF15 Pathway

**DOI:** 10.1155/2016/1092746

**Published:** 2016-03-14

**Authors:** Yajun Yang, Yanjie Su, Dongtao Wang, Yahui Chen, Yuyu Liu, Shiying Luo, Tie Wu, Liao Cui

**Affiliations:** ^1^Department of Pharmacology, Guangdong Key Laboratory for R&D of Natural Drugs, Guangdong Medical University, Zhanjiang, Guangdong 524023, China; ^2^Department of Nephrology, Shenzhen Affiliated Hospital, Guangzhou University of Traditional Chinese Medicine, Shenzhen, Guangdong 518033, China

## Abstract

Decreased bone formation is responsible for the pathogenesis of glucocorticoid- (GC-) induced osteoporosis (GIO), while the mechanism remains to be elucidated. The aim was to investigate how natural antioxidant tanshinol attenuates oxidative stress and rescues impaired bone formation elicited by GC in Sprague-Dawley rats and in C2C12 cells and/or MC3T3-E1 cells. The results showed that tanshinol prevented bone loss and decreased biomechanical characteristics and suppressed reduction of biomarkers related to osteogenesis in GIO rats. Further study revealed that tanshinol reversed decrease of transcription activity of Osterix-luc and rescued impairment of osteoblastic differentiation and bone formation involved in induction of* KLF15* mRNA. Meanwhile, tanshinol diminished inhibition of protein expression of *β*-catenin and Tcf4 and transcription activity of Tcf4-luc induced by GC, especially under conditions of KLF siRNA* in vitro*. Additionally, tanshinol attenuated increase of reactive oxygen species (ROS) generation, phosphorylation of p66^Shc^ expression, TUNEL-positive cells, and caspase-3 activity elicited by KLF15 under conditions of GC. Taken together, the present findings suggest that tanshinol attenuated the decrease of bone formation and bone mass and bone quality elicited by GC involved in KLF15/Wnt signaling transduction and counteracted GC-evoked oxidative stress and subsequent cell apoptosis involved in KLF15/p66^Shc^ pathway cascade.

## 1. Introduction

It is well known that long-term administration of excessive glucocorticoid (GC) leads to glucocorticoid-induced osteoporosis (GIO), a vital risk factor of the increase in the incidence of bone fracture [1]. Bone metabolism disorder has been identified to play a significant vital role in the pathogenesis of GIO [2]. To date, the therapeutic strategy of GIO relies on clinical agents similar to those used for the treatment of postmenopausal osteoporosis, which is distinguished clinically from GIO characterized by the impairment of bone formation [3]. Consequently, most of these drugs for the treatment of GIO show diverse limitations and side effects. Urgently, raising focus on the new findings of bone metabolism related to GC may be beneficial for the development of a novel therapeutic approach of the prevention and treatment of GIO.

Increasing documents demonstrated that oxidative stress triggered by excessive reactive oxygen species (ROS) generation elicits a series of deleterious events in skeletal metabolism, ultimately contributing to the development and progression of osteoporosis [4, 5]. Profiles of genomics analysis showed that varied genes related to oxidative stress are changed in human's osteoblasts exposed to dexamethasone (Dex) [6]. Moreover, investigators have unraveled that Dex can directly or indirectly induce oxidative stress through either inhibition of antioxidant activities or induction of excessive production of ROS [5, 7]. Generally, bone formation needs the vast majority of mature osteoblasts differentiated from preosteoblasts for which Sp7/Osterix is required, while multipotential mesenchymal progenitors differentiate into preosteoblasts for which Runx2/Cbfa1 is required [8]. However, osteoblast is susceptible to oxidative stress, which can cause inhibition of osteoblastic differentiation and increase of cell apoptosis, resulting in impairment of bone formation [9, 10]. Collectively, imbalance of bone metabolism may substantially contribute to bone loss under oxidative stress elicited by GC.

As one member of the shcA family, p66^Shc^ can be activated by phosphorylation of serine 36 in response to a variety of stimuli, which further increases intracellular ROS [11]. The previous report in osteoblasts illustrated substantially that oxidative stress elicited by GC suppresses Wnt signaling, an essential stimulus for osteoblastogenesis, resulting in bone loss [5]. In addition, Kruppel-like factor (KLF) 15, a recently identified glucocorticoid receptor (GR) target gene, is one type of the family of zinc finger-containing transcription factors related to diverse cellular processes including regulation of cell differentiation, angiogenesis, and stem cell fate [12]. Interestingly, KLF15 as a central regulator of stress response interacts with components of the Wnt pathway, resulting in inhibition of *β*-catenin/Tcf-transcriptional activity in cardiac cells [13]. Although Lef/Tcfs mediate canonical Wnt/*β*-catenin signaling in various cell types, Tcf4 are mainly expressed in osteoblasts [14]. Recent evidence showed that expression of KLF15 is increased in osteoblasts exposed to Dex [15]. However, regulatory mechanisms in the downstream of KLF15 pathways in the process of bone metabolism are to be elucidated. Collectively, we ask whether activation of KLF15 elicited by GC causes p66^Sch^-mediated oxidative stress and subsequently promotes cell apoptosis and simultaneously affects regulation of Wnt/Tcf4 signaling of skeletal tissue in the GIO model.

Generally, antioxidants have been considered to have beneficial influences on oxidative stress-associated diseases. Administration of antioxidant was ascertained to exhibit an inhibitory effect on ovariectomy-induced bone loss in rodent model [16]. Previous findings revealed that* D*(+)*β*-3,4-dihydroxyphenyl lactic acid (tanshinol, or named Danshensu) isolated from* Salvia miltiorrhiza* Bunge exerted inhibitory influence on oxidative stress [17]. The previous studies in our team indicated that tanshinol stimulates osteogenesis and depresses adipogenesis, exhibiting a protective action on bone formation in GC treated rats and on bone marrow stromal cells (MSC) exposed to excessive GC [18, 19]. Currently, our previous data confirmed that tanshinol attenuates suppression of osteoblastic differentiation induced by oxidative stress via Wnt/FoxO3a signaling pathway in C2C12 cells and MC3T3-E1 cells, in line with positive control resveratrol, a well-known antioxidant containing polyphenolic acid structure similar to tanshinol [20]. However, the exact signaling mechanism by which tanshinol attenuates impaired bone formation induced by GC has not yet been investigated. Additionally, varied preparation of complex prescription to prevent and treat cardiovascular diseases contains tanshinol, as principal active ingredient in Traditional Chinese Medicine [21]. Consequently, tanshinol may be developed as a potential candidate for prevention and/or treatment of GIO.

Based on the above lines of evidence, in this work presented herein, we will investigate* in vivo* and* in vitro* the notion that regulation of KLF15 pathway cascade may be a new understanding of the mechanisms involved in the pathogenesis of GIO. Meanwhile, we will confirm our hypothesis that tanshinol may exert a protective impact on bone mass and bone strength under oxidative stress elicited by GC and that tanshinol may stimulate regulation of KLF15 pathway cascade, contributing to suppression of oxidative stress and stimulation of bone formation.

## 2. Materials and Methods

### 2.1. Animal Experiments

Four-month-old female Sprague-Dawley rats (200–250 g, *n* = 32) were purchased from the Center of Experiment Animal of Sun Yat-Sen University Ltd., China. Certificate of quality was SCXK (YUE) 2012-0112. The animals were housed in Guangdong Medical College in accordance with the recommendations in the* Guide for the Care and Use of Laboratory Animals* of Guangdong Laboratory Animal Monitoring Institute under the National Laboratory Animal Monitoring Institute of China. All experimental protocols were approved by the Academic Committee on the Ethics of Animal Experiments of the Guangdong Medical College, Zhanjiang, China. Permit number was SYXK (YUE) 2008-0007. All animals were fed with standard chow and had free access to water at optimal temperature ranging from 24°C to 26°C with a humidity level of 70% and a 12-hour light-dark cycle. The animals were randomly assigned to the following four groups (*n* = 8 for each group): Con, standard chow and distilled water; GC, 5 mg prednisone acetate/kg·d; Tan, GC + 16 mg tanshinol/kg·d; Res, GC + 5 mg resveratrol/kg·d. The rats in every group were treated with prednisone acetate in the morning and with other drugs in the afternoon by intragastric administration once a day for 14 weeks. All rats were injected subcutaneously with calcein (10 mg/kg, Sigma Chemical Co., St. Louis, MO) on days 13 and 14 and days 3 and 4 before sacrifice.

### 2.2. Sample Collection and Applications

All rats were sacrificed by cardiac puncture under anesthesia with peritoneal injection of sodium pentobarbital (1.5 mg·kg^−1^ intraperitoneally, Sigma Chemical Co., St. Louis, MO) at the experimental endpoint. Serum was collected by centrifugation for biochemical assays. The right femur was evaluated for the measurements of bone biomechanical characteristics and bone microarchitecture. The proximal metaphysis of right tibia was subjected to undecalcified section for bone histomorphometry. The left femur was used to prepare decalcified section for TUNEL analysis. Bone marrow cells flushed from the left tibia were prepared to measure oxidative stress level as previous method [5]. The left tibia and the 6th lumbar vertebra (LV6) were collected to detect genes expression and proteins level.

### 2.3. Structural and Histological Bone Measurement

Bone trabecular microarchitecture was assessed in the right proximal femur by Micro-CT (SCANCO vivaCT40, Bassersdorf, Switzerland). Briefly, the regions of cancellous bones to be scanned (18 *μ*m/slice) were 1–4 mm distal to the growth plate-epiphyseal junctions. After reconstruction, the following parameters were measured: BV/TV, Tb.N, Tb.Sp, and Tb.Th. For the histomorphometric analysis, the proximal metaphysis of right tibia was fixed in 10% phosphate buffered formalin for 24 h, dehydrated in an ascending ethanol series, and embedded undecalcified in methyl methacrylate. The above tissues were cut into 5 mm sections for von Kossa staining to observe trabecular architectural property and 9 mm sections unstained for assessing the fluorescence labels to analyze bone formation indices such as %L.Pm, MAR, and BFR/TV using the two fluorescent labels. Histomorphometric analysis was performed with the Osteomeasure software (OsteoMetrics, Decatur, GA, USA).

### 2.4. Analysis of Serum Markers

BAP and OCN, as serum markers of bone formation, and OPG, sRANKL, TRAP5b, and OSCAR, as the markers of bone resorption, were measured in rats using commercially available ELISAs (Westang Bio-Tech, Shanghai, China).

### 2.5. Three-Point Bending Test

Mechanical strength of lone bone was determined by a three-point bending test using the testing machinery (MTS, Eden Prairie, Minnesota, USA). The right femurs removed from −20°C were thawed at room temperature and tested with a 1 mm indenter at a speed of 2 mm/minute with a 15 mm span (L). From the load-deformation curve, fracture load (N), elastic load (N), bending energy (N × mm), and stiffness coefficient (N × mm^2^) were obtained by virtue of measurement and calculation.

### 2.6. Cell Culture and Osteoblastic Differentiation Assay

The pluripotent mesenchymal precursor C2C12 cells and preosteoblastic MC3T3-E1 cells were obtained from American Type Culture Collection (ATCC, Manassas, VA, USA). For osteoblastic differentiation, MC3T3-E1 calvarial cells were cultured in *α*-MEM (Gibco BRL, Carlsbad, CA, USA), whereas C2C12 cells were cultured in DMEM (Gibco BRL) containing BMP-2 (100 ng/mL). The osteogenic-induced culture medium was replaced every alternate day. MC3T3-E1 cells and C2C12 cells were maintained in *α*-MEM (Gibco BRL) or DMEM (Gibco BRL) supplemented with 10% FBS, respectively. For determination of ALP activity, cells were stained at day 7 using the BCIP/NBT color development substrate (Nanjing Jiancheng Biotech, China). The stained cellular images were acquired by Eclipse E800 microscope (Nikon, Tokyo, Japan). For analysis of activity of bone formation, MC3T3-E1 cells were stained at day 21 with 2% Alizarin Red S (ARS, pH = 4.2) (Sigma-Aldrich, St. Louis, USA). The images were photographed by microscope (Nikon). The bound ARS was dissolved in a 10% cetylpyridinium chloride monohydrate (CPC) solution (pH 7.0). Absorbance was measured at 545 nm using a microplate reader.

### 2.7. RNA Interference Experiments (si-KLF15) and Overexpression Assay (Ad-KLF15)

C2C12 cells or MC3T3-E1 cells that reached 80–90% confluence were transfected with equal amounts of expression vectors encoding FITC-labeled scrambled sequence control (scrambled, negative control) or gene-specific siRNA of KLF15 (Genepharma, Shanghai, China) in Opti-MEM medium (Invitrogen) using 3 *μ*L Lipofectamine RNAi MAX reagent (Invitrogen) according to the manufacturer's instruction. Cells were transfected with equal amounts of expression adenovirus vectors encoding exogenous KLF15 (Ad-KLF15), mock (empty vectors, negative control), or GFP (Genechem, Shanghai, China) using Lipofectamine LTX (Invitrogen) according to the manufacturer's instruction. After 48 h, transfected cells were induced to osteoblastic differentiation in DMEM containing 5% serum and the medium was replaced periodically as described above. For measurement of mRNA level or luciferase activity, transfected cells were treated with agents as indicated in the Results. Transfection efficiency was monitored on the next day by fluorescence microscopy in the cells transfected with the reporter GFP vectors in serum-free medium. When the transfection efficiency rate was >80%, cells could be used for the following experiments.

### 2.8. Luciferase Assay

The MC3T3-E1 cells and C2C12 cells were plated in 96-well plates at a density of 2 × 10^4^ cells/cm^2^ in triplicate. The cDNAs of Tcf4-luc and Osterix-luc were purchased from Qiagen (Frederick, MD, USA) and transfected using a dual firefly-renilla luciferase reporter assay. After 16 hours, transfected cells were refreshed with fresh media and cultured for an additional 8 hours. Subsequently, cells were serum-starved by culturing in the presence of 2% FBS for 4 hours and treated with or without tanshinol for 1 hour, followed by vehicle control (Con), Dex, and/or related reagents, at indicated concentrations for 24 hours. Then, the cells were lysed with lysis buffer (Promega, Madison, WI, USA) and firefly and renilla luciferase activity using the Dual-Glo Luciferase Assay kit (Promega). The RLU was determined by the ratio of renilla luciferase signal intensity to that of firefly luciferase for normalization.

### 2.9. Quantitative RT-PCR Detection

RNA was extracted from the left tibiae by crushing them in liquid nitrogen and collecting the bone powder in Trifast (Peqlab, Erlangen, Germany). The mononuclear cell fraction was lysed in Trifast (Peqlab). RNA from both C2C12 cells and MC3T3-E1 cells was isolated using Trifast (Peqlab) after washing twice with PBS. RNA isolation was performed according to the manufacturer's protocol. Five hundred nanograms (500 ng) of RNA was reverse-transcribed using Superscript II (Invitrogen, Darmstadt, Germany) and subsequently used for SYBR green-based real-time PCR reactions using a standard protocol (Applied Biosystems, Carlsbad, CA, USA). Primer sequences are summarized in Table 1. Complementary DNA (cDNA) was synthesized, and qRT-PCR was performed on a Stratagene Mx3005P QPCR System (La Jolla, CA, USA). PCR results were analyzed using Opticon Monitor Analysis 2.0 software (Bio-Rad Laboratories, Hercules, CA, USA). Relative mRNA expression was quantified by subtracting the glyceraldehyde-3-phosphate dehydrogenase (GAPDH) threshold cycle (*C*_*t*_) value from *C*_*t*_ value of the genes of interest and expressed as 2^−ΔΔ*C*_*t*_^, as described by the protocol of the manufacturer.

### 2.10. Western Blotting Analysis

For Western blotting, cells were lysed in RIPA buffer containing complete protease inhibitor cocktail. The phosphorylation status of p66^Shc^ was analyzed by immunoblotting in sixth lumbar vertebra or cultured cell lysates, as described previously [22], using a monoclonal antibody recognizing Ser36 phosphorylated p66^Shc^ (Abcam). Protein levels of p66^Shc^ were analyzed using a rabbit polyclonal antibody recognizing Ser36 phosphorylated p66^Shc^ and p66^Shc^ (Abcam). The antibodies recognizing *β*-catenin and Tcf4 were purchased from Cell Signaling Technology. The protein expression was monitored by the measurement of chemiluminescence alterations using Image Station 2000 MM (Eastman Kodak, Rochester, NY, USA).

### 2.11. Other Assays

Intracellular ROS were quantified with 2′,7′-dichlorodihydrofluorescein diacetate (DCFH-DA, Sigma-Aldrich, St. Louis, MO, USA) dye, using bone marrow cells flushed and washed with PBS from tibia, or cultured MC3T3-E1 cells as in previous publication [5]. Glutathione reductase activity (GSR) was assayed with a kit (Beyotime Biotech., Haimen, Jiangsu, China). Apoptosis in sections was measured by TUNEL staining, whereas apoptosis in cultured cells was determined by measuring caspase-3 activity by cleavage of the fluorogenic substrate Ac-DEVD-AFC (Beyotime Biotech., Haimen, Jiangsu, China), as described previously [5].

### 2.12. Statistical Analysis

ANOVA (SPSS 13.0) was used to detect effects of various treatments after establishing equivalency of variances and the notion that the data were normally distributed. Samples were considered normally distributed if *P* > 0.05. Heterogeneity of variance was accepted if *P* > 0.05, and LSD method was used to perform appropriate pairwise comparisons of treatment groups. Unless otherwise stated, the data are presented as the mean ± standard deviation (SD), and the values were considered statistically significant at *P* < 0.05.

## 3. Results

### 3.1. Tanshinol Prevents Bone Loss and Decreased Biomechanical Characteristics in GIO Rats

**Figure 1:**
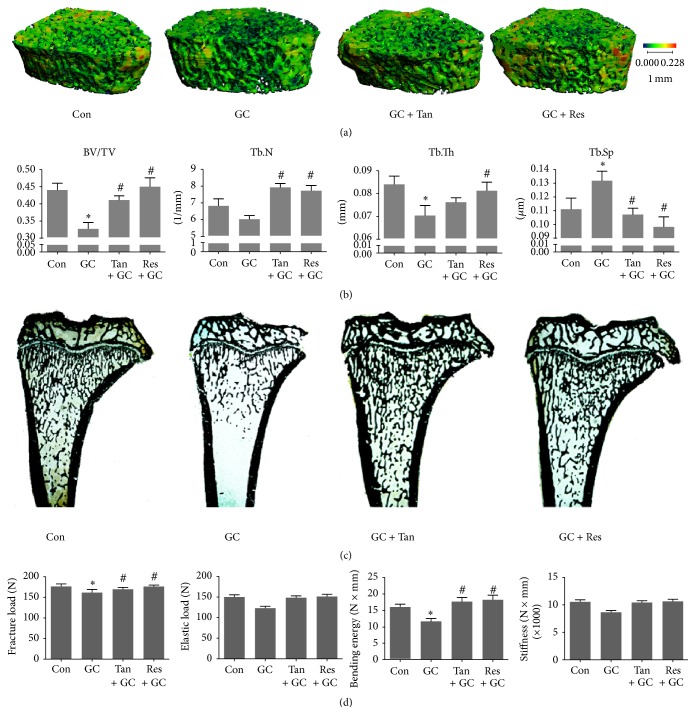
Tanshinol maintains bone microarchitecture and biomechanical properties. Rats were treated with distilled water (Con), prednisone (GC, 5 mg/kg·d), GC plus tanshinol (GC + Tan, 16 mg/kg·d), and GC plus resveratrol (GC + Res, 5 mg/kg·d) for 14 weeks. The following measurements were carried out. (a) Micro-CT reconstruction of the trabecular part of proximal femur of rats. (b) Microarchitectural parameters of proximal femoral spongiosa were measured by Micro-CT machine. (c) von Kossa staining of undecalcified sections of proximal tibia spongiosa of rats. (d) Biomechanics characteristics of femur were determined by three-point bending assay. Data are given as mean ± SD (*n* = 8). ^*∗*^ *P* < 0.05 versus normal control (Con); ^#^ *P* < 0.05 versus GC treatment (GC).

In order to assess the influence of tanshinol on bone architecture and bone quality, we firstly measured structural parameters of trabecular bone using microcomputed tomography (Micro-CT) machine and reconstructed a 3D image of the trabeculae. As is shown in Figures 1(a) and 1(b), rats exposed to GC exhibited impaired bone architecture, as documented by a decrease in the bone volume/tissue volume (BV/TV) and the trabecular thickness (Tb.Th), and an increase of the trabecular separation (Tb.Sp). These data were confirmed by cancellous bone histomorphometric analyses (Figure 1(c)). Tanshinol, however, exerted a significant protective action on bone architecture in GIO rats. To further determine whether the treatment with tanshinol improved biomechanical properties of bone tissue, a three-point bending test was performed on femoral shaft samples. Compared to vehicle controls, the treatment of GC led to the significant reduction of fracture load and bending energy and a trend toward decline in elastic load and stiffness (Figure 1(d)). Expectedly, tanshinol attenuated the deleterious effects of GC on bone biomechanical characteristics. Similar results were obtained in rats exposed to resveratrol. Collectively, tanshinol exhibits a preventive action on bone mass and bone strength, in accordance with protective effect of resveratrol on bone tissue in GIO rats.

### 3.2. Tanshinol Reverses the Imbalance between Bone Formation and Bone Resorption

**Figure 2:**
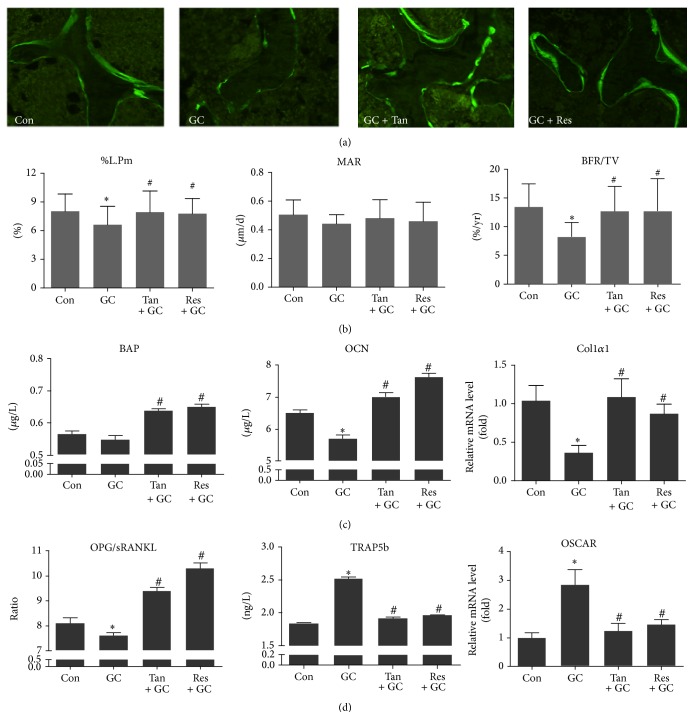
Tanshinol reverses the imbalance between bone formation and bone resorption. Procedures of treatment in rats were carried out as described in Figure 1, and determinations were executed as follows. (a) Fluorescent micrographs of dual calcein labeling in tibia of rats. (b) Histomorphometric quantitative analysis of dynamic parameters of %L.Pm, MAR, and BFR/TV used as key indicators of bone-forming capacity in tibia spongiosa of rats. (c) Biomarkers of the bone formation including serum BAP, serum OCN, and* Col1α1* mRNA level were measured using ELISA assay and qRT-PCR, respectively. (d) Ratio of serum OPG/RANKL reflecting changes of bone turnover and the biomarkers related to bone resorption including serum TRAP5b and* OSCAR* mRNA level of bone tissue. Data are given as mean ± SD (*n* = 8). ^*∗*^ *P* < 0.05 versus normal control (Con); ^#^ *P* < 0.05 versus GC treatment (GC).

We next set out to investigate the protective effect of tanshinol on bone tissue via improvement of metabolic imbalance between bone formation and bone resorption elicited by long-term excessive GC in rats. Dynamic alteration of bone formation was indicated by calcein double-labeled trabeculae in the distal femur. As was shown in the representative histologic images, larger space between the calcein labels and stronger fluorescence intensity were observed in bone sections of rats in Con, GC + Tan, and GC + Res groups than those of GC group (Figure 2(a)). The evidence of decreased bone formation was also demonstrated by a reduced percent labeled perimeter (%P-L.Pm) and the mineral apposition rate (MAR), as well as the bone formation rate (BFR/TV). MAR as an index of the capacity of individual osteoblasts to form bone mineral was about 34% decreased in GIO rats compared with control rats. Meanwhile, BFR/TV determined by the number and function of osteoblasts exhibited an approximate 44% decrease in GIO rats compared with control rats (Figure 2(b)). In line with the histological data, the deleterious effects of GC on bone metabolism were also consolidated by the alterations of biomarkers of bone turnover. We confirmed that GC resulted in the decrease of biomarkers of the bone formation, including serum bone specific alkaline phosphatase (BAP), serum osteocalcin (OCN), and* collagen I α1 (Col1α1)* mRNA level of bony tissue (Figure 2(c)). Contrarily, GC stimulated the increase of the biomarkers related to bone resorption including serum TRAP5b and* OSCAR* mRNA level of bone tissue. Furthermore, GC contributed to high bone turnover rate reflected by decreased serum OPG/RANKL ratio (Figure 2(d)). Encouragingly, tanshinol showed a capacity to reverse the deleterious impacts of bone turnover elicited by GC, as effectively as resveratrol (Figure 2). In brief, these lines of evidence confirm that tanshinol can promote the increase of bone formation and simultaneously prevent bone resorption.

### 3.3. Tanshinol Stimulates Wnt-Mediated Osteoblast Differentiation Involved in KLF15

**Figure 3:**
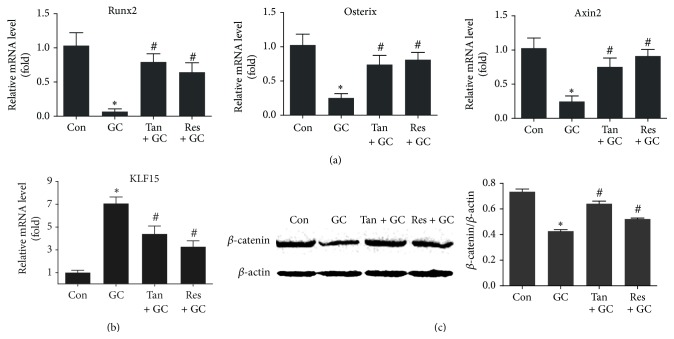
Tanshinol protects osteoblastic differentiation against GC involved in Wnt signaling and KLF15 transcriptional factor. Rats were treated as in Figure 1, and measurements were made as follows. (a) mRNA levels of* Runx2* gene and* Osterix* gene which contribute to osteoblast differentiation and of* Axin2* gene (an indicator of Wnt pathway) were determined by qRT-PCR assay in long bone of rats. (b) mRNA levels of* KLF15* gene were detected by qRT-PCR assay in long bone of rats. (c) Expression of *β*-catenin protein (a key molecule of canonical Wnt signaling) in the left tibia was measured by Western blot method. Representative figure was shown on the left panel, and quantification is shown on the right panel. Data are given as mean ± SD (*n* = 3). ^*∗*^ *P* < 0.05 versus normal control (Con); ^#^ *P* < 0.05 versus GC treatment (GC).

It is well known that skeletal structural fragility results from impaired osteoblastic differentiation and subsequent bone formation. According to the results of qRT-PCR, mRNA expression of runt-related transcription factor 2 (*Runx2*) and* Osterix* gene, which are characteristic early markers of osteogenesis, was hindered by GC treatment (Figure 3(a)). Strikingly, mRNA expression of KLF15 transcription factor, a direct target of GR, was increased in bone tissue of rats treated by GC (Figure 3(b)). Additionally, mRNA expression of* Axin2*, an indicator of Wnt pathway, was inhibited by GC treatment, in line with the evidence that expression level of *β*-catenin protein (a key molecule of canonical Wnt signal transduction) was inhibited by GC using Western blot assay (Figures 3(a) and 3(c)). Expectably, tanshinol blocked the decrease of the two vital biomarkers of osteogenesis and the two key proteins of Wnt signaling, while counteracting the increased expression of KLF15, just like resveratrol. Taken together, these findings suggest that tanshinol rescues the inhibition of Wnt/*β*-catenin signaling in charge of bone formation and exerts an inhibitory action on activation of KLF15 pathway elicited by GC.

### 3.4. Tanshinol Inhibits Oxidative Stress Mediated by p66^Shc^ in Response to Dex and Ameliorated Cell Apoptosis

**Figure 4:**
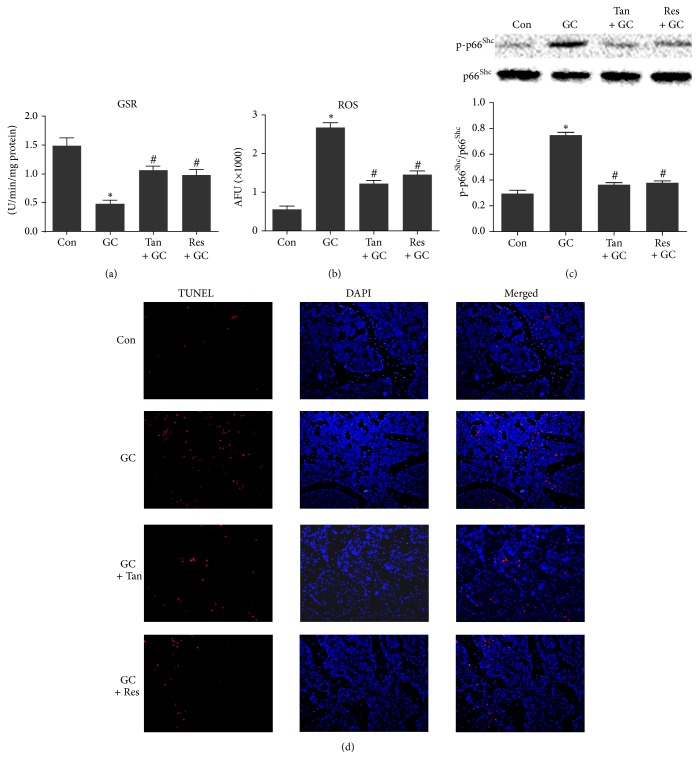
Tanshinol inhibits oxidative stress mediated by p66^Shc^ and hampers cellular apoptosis in response to GC. Rats were treated as in Figure 1, and determinations were made as follows. (a) GSR (a critical endogenous antioxidant) activity in bone marrow cells flushed from tibia was assayed using colorimetric assay (*n* = 4). (b) Oxidative stress indicated as amount of ROS generation in bone marrow cells was estimated by DCFH-DA assay, and the fluorescence intensity of DCF was quantified using Image Station 2000 MM assay (*n* = 4). (c) Phosphorylated p66^Shc^ (a significant mediator of amplification of oxidative stress) in lysates from vertebrae was detected by Western blot assay (*n* = 3). Representative figure was shown on the upper panel, and quantification is shown on the lower panel. (d) Apoptosis in sections was measured by TUNEL staining using fluorescent microscope (*n* = 3). Data are given as mean ± SD. ^*∗*^ *P* < 0.05 versus normal control (Con); ^#^ *P* < 0.05 versus GC treatment (GC).

Cellular redox status of skeletal tissue plays an important role in intracellular signaling pathways during the process of bone metabolism. To evaluate oxidative stress level, we measured the accumulation of intracellular reactive oxygen species (ROS) level and activity of intracellular GSR. As shown in Figures 4(a) and 4(b), the level of ROS generation was increased remarkably, and the activity of GSR was decreased in bone tissue of rats exposed to Dex. Next, we investigated the phosphorylation of p66^Shc^, an adapter protein that amplifies mitochondrial ROS generation and stimulates apoptosis. Evidently, expression level of the phosphorylation of p66^Shc^ was elevated in vertebral lysates of rats treated with GC (Figure 4(c)). Additionally, apoptosis analysis showed that TUNEL-positive cells were increased in femoral cancellous bone from GIO rats (Figure 4(d)). Remarkably, tanshinol exerted an antioxidative stress effect to protect bone tissue against GC, as effectively as resveratrol. These lines of evidence revealed that tanshinol attenuates oxidative stress responsible for cell apoptosis elicited by GC via regulation of ROS/p66^Shc^ pathway in GIO rats.

### 3.5. Tanshinol Reversed Impaired Osteogenesis Linked to Inhibition of KLF15 in Response to GC

**Figure 5:**
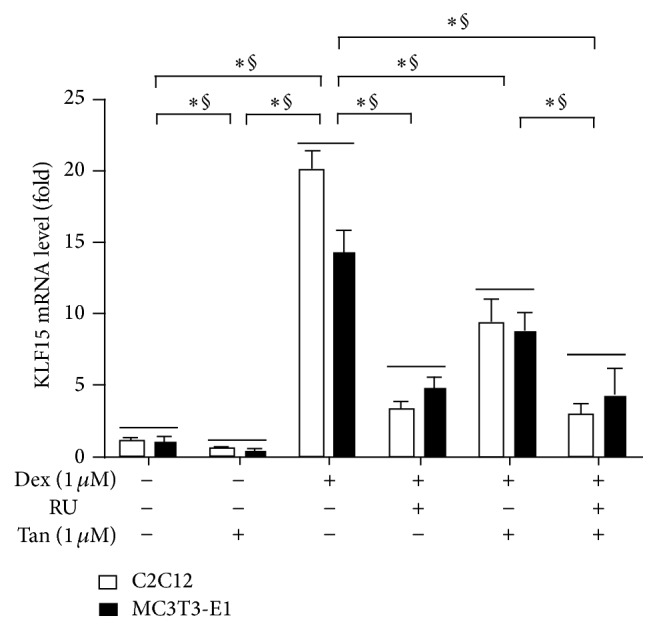
Tanshinol regulates expression of* KLF15* gene under condition of Dex involving glucocorticoid receptor. C2C12 cells and MC3T3-E1 cells were treated with Dex and/or RU486 (RU, a direct target of glucocorticoid receptor) in the presence or absence of Tan for 12 h; mRNA expression of* KLF15* gene was measured by qRT-PCR. Values are means ± SD of at least three independent experiments. ^*∗*^ *P* < 0.05 versus indicated group in C2C12 cells; ^§^ *P* < 0.05 versus indicated group in MC3T3-E1 cells.

Encouragingly, our data* in vivo* indicated that tanshinol counteracted the activation of KLF15 transcription factor, a direct target of GR. To ask whether tanshinol hinders GC-induced negative regulatory role of KLF15 on bone formation* in vitro*, we further detected alterations of* KLF15* mRNA using qRT-PCR assay. The results showed that tanshinol could lead to downregulated expression of* KLF15* gene, while Dex could significantly induce expression of* KLF15* gene in C2C12 cells and MC3T3-E1 cells. However, the Dex-induced expression of KLF15 mRNA was hampered by RU486, a GR antagonist. Interestingly, tanshinol counteracted increase of KLF15 expression elicited by Dex in the two cells, especially in the presence of RU486 (Figure 5). The data may provide a clue to understanding the molecular mechanism of the protective effect of tanshinol on bone metabolism concerning regulation of KLF15.

**Figure 6:**
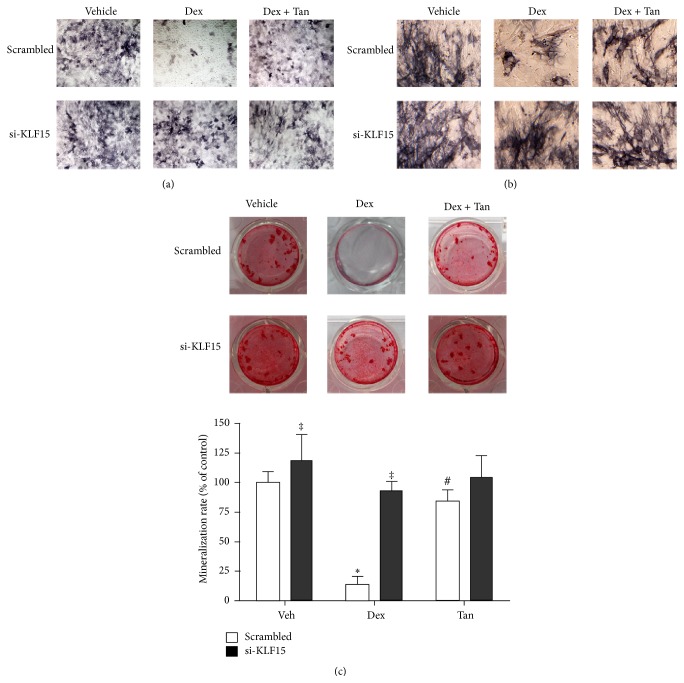
Tanshinol counteracts inhibition of osteoblastic differentiation and bone formation elicited by Dex in connection with downregulation of KLF15. C2C12 cells and MC3T3-E1 cells were transfected with KLF15 siRNA for 18 h, followed by DMEM medium supplemented with Dex in the presence or absence of tanshinol for 7 days. (a) Capacity of osteoblastic differentiation in C2C12 cells was determined by using ALP staining. (b) Capacity of osteoblastic differentiation in MC3T3-E1 cells. Original magnification (×100) in representative microscopic images. (c) Effects of knockdown of KLF15 on activity of bone formation. MC3T3-E1 cells were treated with KLF15 siRNA for 18 h, followed by Dex treatment with or without tanshinol for 21 days. Mineralization activity with the indicated treatments was stained using Alizarin Red S at day 21. Original magnification (×100) in representative microscopic images (upper panel). Quantitative determination was carried out by CPC solution (pH 7.0) (lower panel). Vehicle: vehicle control (Veh). Values are means ± SD of at least three independent experiments. ^*∗*^ *P* < 0.05 versus vehicle control; ^#^ *P* < 0.05 versus GC treatment; ^‡^ *P* < 0.05 versus corresponding scrambled control.

Since little is known about whether GR-dependent KLF15 impairs osteogenesis, we next examined the effects of knockout of* KLF15* gene on the capacity of osteoblastic differentiation and bone formation in pluripotent mesenchymal precursor C2C12 cells and preosteoblastic MC3T3-E1 cells transfected transiently with siRNA oligonucleotides targeting KLF15. Based on the evidence of ALP staining, Dex-elicited decreased capacity of osteoblastic differentiation was blocked by KLF15 siRNA in the two cells (Figures 6(a) and 6(b)), as well as activity of bone formation measured by Alizarin Red S staining (Figure 6(c)). More importantly, the two cells exposed to tanshinol alone or in association with KLF15 siRNA could maintain good capacity of osteogenesis under conditions of Dex (Figure 6).

**Figure 7:**
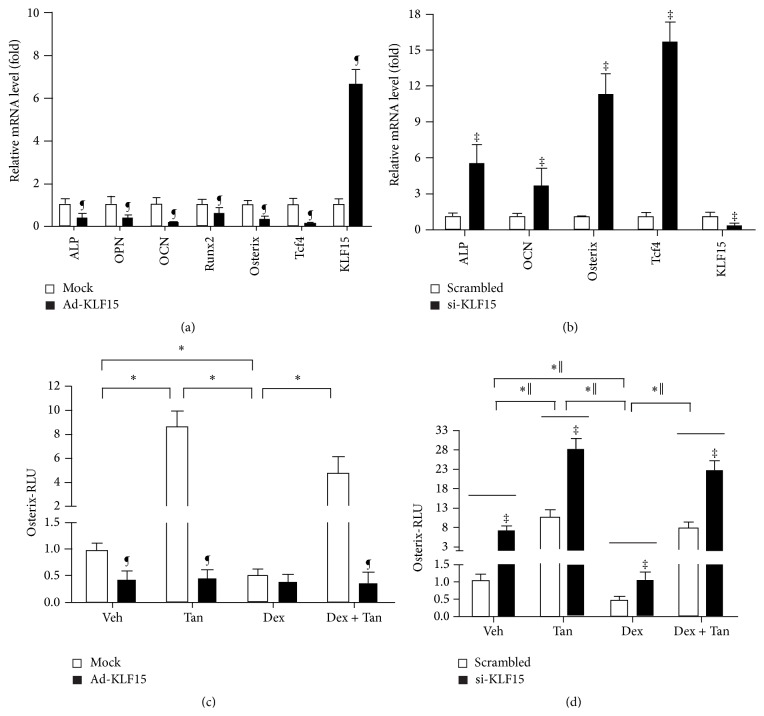
Regulation of differential genes by KLF15 and involvement of Osterix in protective effect of tanshinol on bone formation. (a) MC3T3-E1 cells were infected with recombinant adenovirus Ad-KLF15 for 4 days. mRNA expression of bone formation-related genes and KLF15 gene was measured by qRT-PCR. (b) MC3T3-E1 cells transfected with KLF15 siRNA for 18 h. mRNA expression of bone formation-related genes and KLF15 gene was measured by qRT-PCR. (c) MC3T3-E1 cells were infected with Osterix-luc reporter plasmid in combination with recombinant adenovirus Ad-KLF15 or mock (noninfection). (d) MC3T3-E1 cells were cotransfected with the Osterix-luc reporter plasmid in combination with KLF15 siRNA or the scrambled sequence. Luciferase activity assays were explored using the Dual-Luciferase Reporter Assay System as described under Section 2.8 in Materials and Methods. The data represent mean ± SD of luciferase relative luminescence units (RLU) normalized to corresponding renilla luciferase activity (triplicates). ^*∗*^ *P* < 0.05 versus indicated group in cells exposed to empty vector (mock) or scrambled control; ^‖^ *P* < 0.05 versus indicated group in cells exposed to KLF15 siRNA or recombinant adenovirus Ad-KLF15; ^¶^ *P* < 0.05 versus corresponding mock; ^‡^ *P* < 0.05 versus corresponding scrambled control.

Next, we investigated the alteration in the gene expression profile related to osteogenesis in MC3T3-E1 cells transfected with the adenovirus-mediated exogenous expression of KLF15 or KLF15 siRNA. Surprisingly, the exogenous expression of KLF15 weakened induction of mRNA expression of* ALP*,* OPN*,* OCN*,* Runx2*,* Osterix* (a transcription factor required for osteoblast differentiation and bone formation), and* Tcf4* (an effector of downstream of Wnt signaling), while promoting increase of KLF15 mRNA level by approximately 7-fold (Figure 7(a)). In clear contrast, KLF15 siRNA caused induction of* ALP*,* OCN*,* Osterix*, and* Tcf4* expression, while it resulted in reduction of KLF15 mRNA level (Figure 7(b)). We then focused on Osterix and asked whether Dex-elicited reduction of osteogenesis was mediated by KLF15 using Osterix-luc reporter plasmid in MC3T3-E1 cells exposed to exogenous KLF15 or knocking down the expression of KLF15. Overexpression of KLF15 could repress the relative luminescence units (RLU) of Osterix-luc but Dex showed no influence on RLU of Osterix-luc. Meanwhile, Dex contributes to decrease of RLU of Osterix-luc and tanshinol could attenuate the inhibitory effect of Dex on RLU of Osterix-luc in cells treated with mock (empty vector) (Figure 7(c)). Reversibly, KLF15 siRNA contributed to increase of RLU of Osterix-luc. Moreover, tanshinol counteracted decreased RLU of Osterix-luc elicited by Dex in MC3T3-E1 cells treated with or without KLF15 siRNA (Figure 7(d)). Taken together, these data revealed that transcription activity of Osterix-luc is negatively regulated by KLF15 in response to GC, which may contribute to impaired capacity of osteoblastic differentiation and the following bone formation.

### 3.6. Tanshinol Counteracts GC-Elicited Oxidative Stress and Cell Apoptosis Involved in KLF15/p66^Shc^ Pathway Cascade

**Figure 8:**
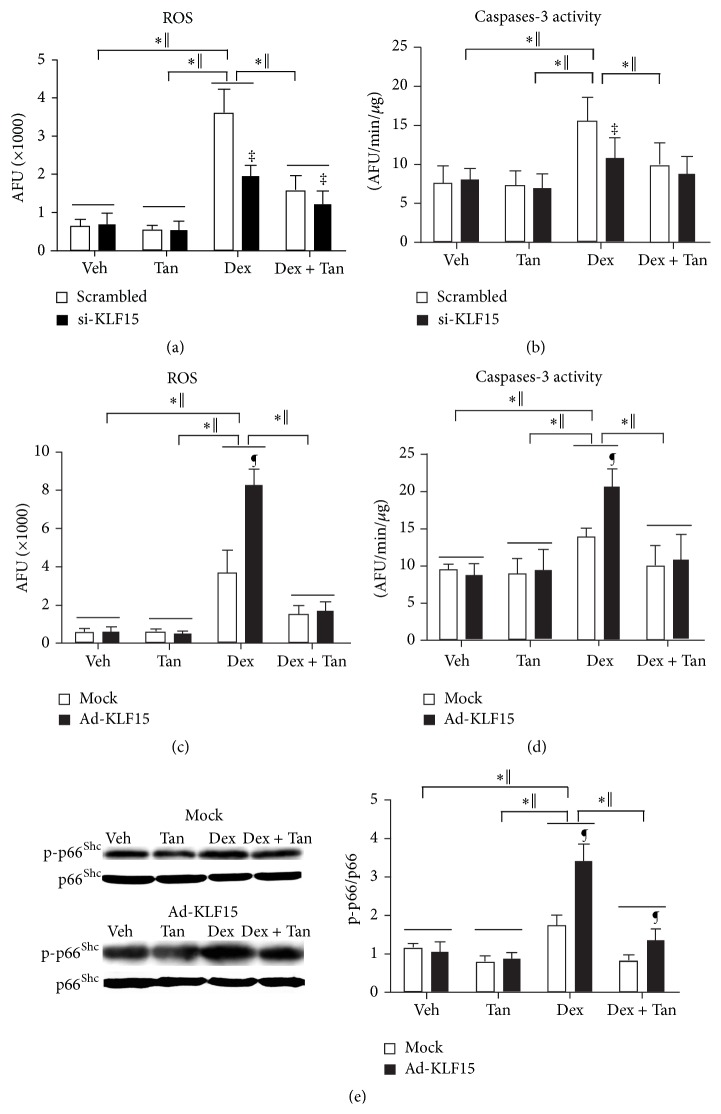
Tanshinol counteracts GC-induced oxidative stress and caspase-3-dependent apoptosis linked to phosphorylation of p66^Shc^. MC3T3-E1 cells were transfected with KLF15 siRNA or recombinant adenovirus Ad-KLF15 in the presence or absence of Dex and/or tanshinol, and measurements were explored as follows. ((a) and (c)) ROS level indicated oxidative stress status was analyzed by DCFH-DA probe. ((b) and (d)) Cellular apoptosis was detected by caspase-3 activity. (e) Phosphorylated p66^Shc^ in MC3T3-E1 cells exposed to Ad-KLF15 and mock was detected by Western blot assay (left panel). Representative figure was shown on the left panel, and quantification is shown on the right panel. Bars indicate mean ± SD of triplicate determinations. ^*∗*^ *P* < 0.05 versus indicated group in cells exposed to empty vector (mock) or scrambled control; ^‖^ *P* < 0.05 versus indicated group in cells exposed to KLF15 siRNA or recombinant adenovirus Ad-KLF15; ^¶^ *P* < 0.05 versus corresponding mock; ^‡^ *P* < 0.05 versus corresponding scrambled control.

Concerning the evidence that phosphorylation of p66^Shc^ in bone is associated with increased cell apoptosis* in vivo*, as well as evidence that p66^Shc^ amplifies ROS generation in mitochondria [5] and thereby promotes apoptosis, we investigated whether tanshinol could protect osteoblasts from oxidative stress and subsequent apoptosis elicited by GC involved in regulation of KLF15/p66^Shc^ pathway cascade. In MC3T3-E1 cells exposed to overexpression of KLF15 alone or KLF15 siRNA alone, level of ROS generation and cleavage activity of caspase-3 kept unchanged, as well as expression of p-p66^Shc^ protein. However, Dex provoked excessive ROS generation, higher cleavage activity of caspase-3, and more expression of p-p66^Shc^ protein, in various degrees which were strengthened by treatment with overexpression of KLF15; however, ROS generation and caspase-3 activity induced by Dex could be weakened by KLF15 siRNA. Additionally, tanshinol attenuated ROS generation, caspase-3 activity, and p-p66^Shc^ expression elicited by Dex in MC3T3-E1 cells, especially in the presence of KLF15 siRNA (Figure 8). Therefore, it is likely that tanshinol protects osteoblasts against Dex in connection with suppression of induction of KLF15 which may cause phosphorylation of p66^Shc^ contributing to accumulation of ROS generation and subsequent cell apoptosis.

### 3.7. Tanshinol Counteracts Negative Regulation of Wnt Signaling by Dex Linked to KLF15/Tcf4 Pathway

**Figure 9:**
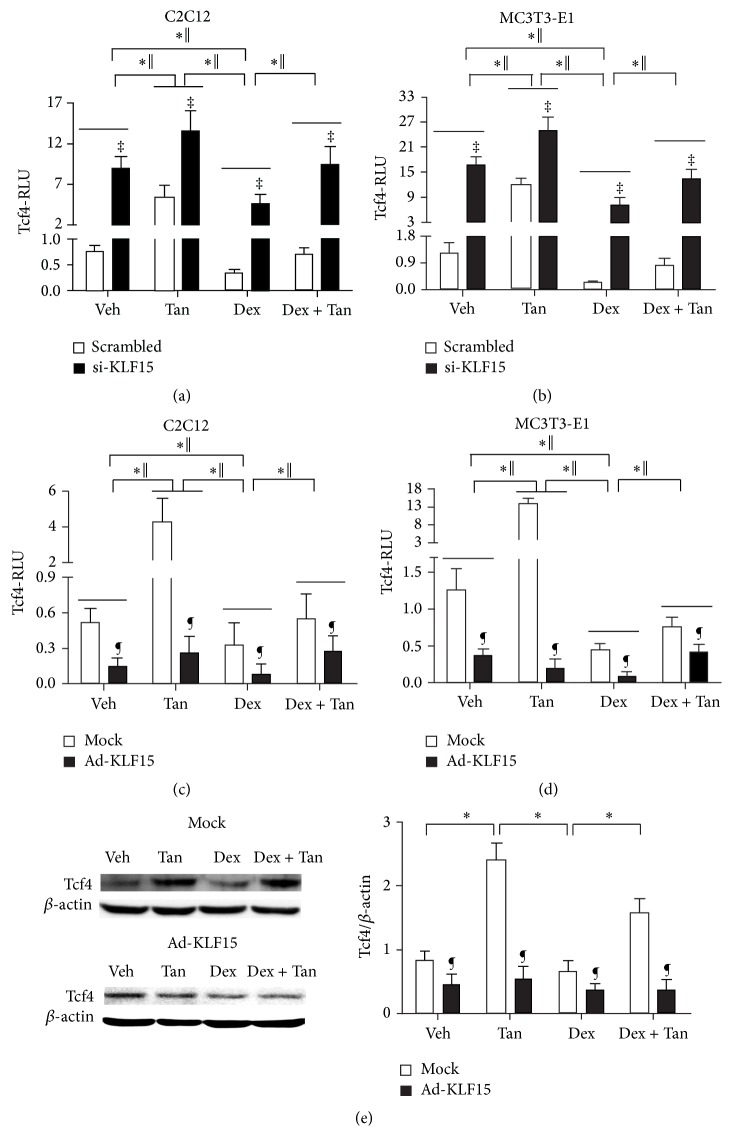
Tanshinol attenuates downregulation of canonical Wnt signaling elicited by Dex associated with regulation KLF15. ((a) and (b)) C2C12 cells or MC3T3-E1 cells were cotransfected with the Tcf4-luc or FoxO3a-luc reporter plasmid in combination with KLF15 siRNA or the scrambled sequence. ((c) and (d)) C2C12 cells or MC3T3-E1 cells were infected with the FoxO3a-luc or Tcf4-luc reporter plasmid in combination with recombinant adenovirus Ad-KLF15 or mock (noninfection). Luciferase activity assays were explored using the Dual-Luciferase Reporter Assay System as described under Section 2.8 in Materials and Methods. The data represent mean ± SD of luciferase relative luminescence units (RLU) normalized to corresponding renilla luciferase activity (triplicates). (e) Tcf4 (a requisite mediator for downstream effector Tcf of canonical Wnt pathway contributing to bone formation) in MC3T3-E1 cells exposed to Ad-KLF15 and mock was detected by Western blot assay. Representative figure was shown on the left panel, and quantification is shown on the right panel. Bars indicate mean ± SD of triplicate determinations. ^*∗*^ *P* < 0.05 versus indicated group in cells exposed to empty vector (mock) or scrambled control; ^‖^ *P* < 0.05 versus indicated group in cells exposed to KLF15 siRNA or recombinant adenovirus Ad-KLF15; ^¶^ *P* < 0.05 versus corresponding mock; ^‡^ *P* < 0.05 versus corresponding scrambled control.

To further elucidate the underlying mechanism for tanshinol to counteract KLF15-mediated reduction of osteogenesis under conditions of GC, we observed transcription activity of Tcf4-Luc and expression of Tcf4 protein in MC3T3-E1 cells and/or C2C12 cells treated with KLF15 siRNA or exogenous KLF15. The results showed that the RLU of Tcf-luc and expression of Tcf4 protein were evaluated by virtue of KLF15 siRNA in the two cell lines, while they were declined owing to overexpression of KLF15. Interestingly, the promoting role of tanshinol on Tcf-luc seemed to be more significant in the two cells transfected with KLF15 siRNA than those treated with tanshinol alone, whereas overexpression of KLF15 partly neutralized this promoting effect of tanshinol on Tcf-luc and expression of Tcf4 protein (Figures 7(a) and 7(c)). Moreover, tanshinol ameliorated the decreased RLU of Osterix-luc elicited by Dex in the two cells, especially in the presence of KLF15 siRNA (Figure 9). Therefore, the data suggested that tanshinol arrests downregulation of Wnt pathway responsible for bone formation under conditions of Dex involved in KLF15/Tcf4 pathway.

## 4. Discussion

Increasing well-documented evidence highlights the role of oxidative stress in the development and progression of osteoporosis [23–25]. We confirmed herein that oxidative stress elicited by GC contributes to bone loss and impaired bone strength and this might be hampered by antioxidants. Previous evidence in our team demonstrated that tanshinol as a natural antioxidant exhibited the potential to promote osteoblastic differentiation and bone formation, contributing to a strongly preventive effect on GIO [18, 20], as efficiently as resveratrol [26, 27]. In the present work, tanshinol counteracts reduction of trabecular parameters, impaired biomechanical characteristics, and imbalance of bone turnover parameters in the experimental model of GIO, respectively, just like resveratrol in light of the previous evidence [26, 27]. The findings herein reveal that tanshinol diminishes the deleterious effects of GC on bone quality involved in inhibiting downregulation of Wnt signaling responsible for osteogenic differentiation and bone formation and suppressing activation of ROS/p66^Shc^ pathway cascade for oxidative stress and cell apoptosis under conditions of GC. Particularly, based on the evidence from siRNA interference and overexpression methods, we firstly elucidated in this study that tanshinol ameliorates induction of KLF15 transcription factor elicited by GC which may give rise to activation of p66^Shc^ pathway and lead to arrest of Wnt signaling during osteoblastic differentiation and bone formation.

Tanshinol, consisting of polyphenolic hydroxyl groups similar to resveratrol, exhibits the inhibitory action on oxidative stress* in vitro* [20, 28, 29]. In the present paper, we found that tanshinol exerted a serial of antioxidative stress actions in bone tissue of GIO rats and/or in MC3T3-E1 cells exposed to Dex, including reduction of ROS generation, increase of GSR activity, and inhibition of phosphorylated p66^Shc^ in GIO rats, as effectively as antioxidants like resveratrol [30]. Similarly, tanshinol could counteract increase of cell apoptosis measured by TUNEL assay* in vivo* and determined by cleavage activity of caspase-3* in vitro*. Interestingly, the indexes of oxidative stress and cell apoptosis, including expression of phosphorylated p66^Shc^, ROS generation, and caspase-3 activity, showed at low level under normal conditions in MC3T3-E1 cells in the absence of Dex, and tanshinol showed no effects in cells treated with either overexpression of KLF15 or KLF15 siRNA. Surprisingly, the indexes of oxidative stress and cell apoptosis mentioned above are strongly induced and activated in response to Dex in MC3T3-E1 cells, especially treated with overexpression of KLF15, and tanshinol could attenuate the activation of oxidative stress and cell apoptosis, in synergy with KLF15 siRNA. It is a plausible mechanism that ROS generation as a cellular metabolic activity maintains redox balance between oxidants and antioxidants for homoeostasis in general, but it can be overwhelmingly increased under conditions of oxidative stress elicited by Dex [31], and tanshinol as a natural antioxidant can exert a significant inhibitory effect on excessive ROS generation and can delay a series of deleterious events to damage molecule, tissues, and organs. p66^Shc^ is a robust marker of oxidative stress, known as a sensor as well as amplifier of oxidative stress, because p66^Shc^ can promote ROS generation by virtue of activation and further increase of intracellular ROS [32]. Furthermore, p66^Shc^ activated by phosphorylation on serine 36 (S36) is an event on which the proapoptotic function of p66^Shc^ depends [33]. The p66^Shc^ protein mediates oxidative stress-related injury in multiple tissues [32]. In the present study, bone tissue of GIO rats showed significant increase of expression of p66^Shc^ phosphorylation and ROS level, as well as cell apoptosis, which could be hampered by tanshinol and its implications for inhibition of the prevention and treatment of osteoporosis elicited by oxidative stress under conditions of GC.

Oxidative stress significantly leads to harmful consequence of increased osteoblastic apoptosis seen in GIO rats, as illustrated by the finding of increased osteoblast survival following the administration of tanshinol or resveratrol. The current findings are in agreement with our previous* in vitro* findings, in which tanshinol hindered inhibition of proliferation, cell cycle arrest, and increase of apoptosis in C2C12 cells under oxidative stress [20]. Meanwhile, oxidative stress hampers osteogenesis by virtue of inhibition of Wnt signaling [4, 23]. In skeletal tissue, suppression of Wnt signaling by ROS may lead to reduction of the differentiation and survival of osteoblasts and ultimately decreased bone formation [23]. As indispensable signaling of osteoblastic differentiation and bone formation, Wnt pathway regulates expressions of target genes related to osteogenesis (including ALP, OCN, and Runx2) via Tcf4 transcription factor which can be activated by association with *β*-catenin [34, 35]. Moreover, Osterix is an osteoblast-specific transcription factor required for osteoblast differentiation, acts as downstream of Runx2 to induce mature osteoblasts, and attenuates osteoblast proliferation via inhibition of Wnt pathway owing to feedback during osteoblastic differentiation and bone formation [36]. In the present study, both Osterix-mediated and Tcf4-mediated transcription were hindered by Dex* in vitro*, in accordance with previous publications [5, 37]. Additionally, tanshinol could attenuate decrease of transcriptional activity of Osterix and Tcf4 elicited by GC, as well as expression of Tcf4 protein, indicating that tanshinol can suppress inhibition of osteogenesis involved in downregulation of Wnt signaling. Thus, the increased anabolic efficacy of tanshinol may be at least in part due to suppression of the oxidative stress that obstructs osteogenesis and osteoblastic survival in GIO rats, in line with our previous* in vitro* finding [20].

Recently, findings suggest a regulatory impact of KLF15 transcription factor on downregulation of Wnt/*β*-catenin pathway in cardiac homeostasis [13]. KLF15 transcription factor as a direct GR target gene exhibits an extensive role in pathophysiologic progression of diverse disease in varied organs, such as heart fibrosis [38], cardiac lipid metabolism [39], hepatic gluconeogenesis [40], chronic kidney disease [41], muscle wasting [42], and airway hyperresponsiveness [43]. Strikingly, we provide the first evidence herein that induction of KLF15 expression elicited by GC attenuates osteogenesis involved in suppression of Wnt/*β*-catenin pathway and the following Tcf4-dependent transcriptional activation in C2C12 cells and MC3T3-E1 cells. Firstly, the levels of* KLF15* mRNA increase in the GIO rats, and induction of* KLF15* mRNA is observed in C2C12 cells and MC3T3-E1 cells exposed to Dex, in accordance with the previous evidence that Dex promotes expression level of* KLF15* mRNA in primary osteoblasts [15]. Moreover, our data also illustrated a phenomenon of downregulation of* KLF15* mRNA in C2C12 cells and MC3T3-E1 cells treated with RU486, an antagonist of GR, indicating that GC is responsible for the activation of KLF15 transcription factor. In addition, KLF15 is likely to be implicated in the regulation of multiple genes, including those participating in glucose transport [44], energy homeostasis [42], podocyte differentiation [45], adipogenesis [46, 47], and so forth. In a previous study,* KLF15* mRNA was found to increase during the process of osteoblastic differentiation in MSC, but the mechanism remains unclear [48]. However, further detection under conditions of siRNA interference or overexpression of KLF15 may help to clarify the role of KLF15 transcription factor in regulating signaling transduction associated with osteogenesis. Notably, we show here that overexpression of KLF15 results in significantly decreased genes related to osteogenic differentiation and the components of Wnt pathway, which were reversely upregulated by virtue of siRNA interference of KLF15, suggesting that KLF15 may be a vital protein to regulate osteogenesis. Taken together, our study on C2C12 cells and/or MC3T3-E1 cells treated with knockout of KLF15 gene or overexpression of KLF5 further confirmed that tanshinol may protect against GC-elicited repression of osteogenesis involved in KLF15 pathway.

In conclusion, our findings provide evidence for a novel target of KLF15 transcription factor for skeletal niche in GIO model and reveal a preventive effect of tanshinol on bone tissue involved in inhibition of oxidative stress and subsequent osteoblastic apoptosis via ROS/p66^Shc^ pathway cascade and reduction of impaired bone formation via Wnt/*β*-catenin/Tcf4 signaling transduction. Furthermore, notwithstanding the beneficial influence of resveratrol on bone, it has not been developed as a therapeutic agent in clinical use, only as an extensive tool for the purpose of research by virtue of severe defects in poor solubility and pharmaceutical preparation property [49]. Contrarily, as a water-soluble compound, tanshinol is commonly used as an indicator of diverse complex prescription for quality control in Traditional Chinese Medicine; thus the clinical use of tanshinol will hold promise for an effective and safe candidate for the prevention and treatment of GIO. Our results strongly suggest that tanshinol with optimized pharmacological properties could be developed for therapeutic use in human.

## Figures and Tables

**Table 1:** Primers sequences for real-time PCR analyses of gene expression.

Genes	Species	Primer sequences	
		Forward primer (5′-3′)	Reverse primer (5′-3′)
*Axin2*	Rat	AGTCAGCAGAGGGACAGGA	CTTGGAGTGCGTGGACACTA

*Col1α1*	Rat	TGACCTCAAGATGTGCCACT	GGGAGTTTCCATGAAGCCAC

*OSCAR*	Rat	CTGGTCATCAGTTCCGAAGG	CTATGATGCCCAAGCAGATG

*Tcf4*	Mouse	CCAATCACGACAGGAGGATT	TGATGCTTTGAGCTGTGGAG

*ALP*	Mouse	AACCCAGACACAAGCATTCC	GCCTTTGAGGTTTTTGGTCA

*OPN*	Mouse	TCTCCTTGCGCCACAGAATG	TCGGTACTGGTGTACCTGCT

*OCN*	Mouse	CCATGAGGACCCTCTCTCTGC	AAACGGTGGTGCCATAGATGC

*Runx2*	Rat	ATTCCTGTAGATCCGAGCACCA	TACCTCTCCGAGGGCTACAACC
	Mouse	TACCAGCCACCGAGACCAA	AGAGGCTGTTTGACGCCATAG

*Osterix*	Rat	AGCTCTTCTGACTGCCTGCCTAGT	TTGGGCTTATAGACATCTTGGGGT
	Mouse	AGCGACCACTTGAGCAAACAT	GCGGCTGATTGGCTTCTTCT

*KLF15*	Rat	TCCTCCAACTTGAACCTGTC	CTTGGTGTACATCTTGCTGC
	Mouse	CAAGAGCAGCCACCTCAAG	GACACTGGTACTGCTTCACA

*GAPDH*	Rat	CCATCATGAAGTGTGACGTG	ACATCTGCTGGAAGGTGGAC
	Mouse	ATTGTCAGCAATGCATCCTG	ATGGACTGTGGtcATGAGCC
